# Supplementary material for: Cloud BioLinux: pre-configured and on-demand bioinformatics computing for the genomics community
Source: BMC Bioinformatics. 2012 Mar 19;13:42. doi: 10.1186/1471-2105-13-42 (PMC3372431; doi:10.1186/1471-2105-13-42)
Supplement: Additional file 1 — Supplementary 1 Cloud BioLinux software documentation in the form of a mini, self-contained website. Users need to download and uncompress the .zip file, and open through a web browser the "index.html" file available on the main directory. (ZIP 1823 kb). [file 1471-2105-13-42-S1.ZIP › Cloud-BioLinux-Package-Documentation/docs/dotur.html]

Bio-Linux Software Documentation Pages

Back to search form

## dotur

|  |  |
| --- | --- |
| Name | dotur |
| Description | **DOTUR** expects a distance matrix created from sequence alignment data and assigns sequences to operational taxonomic units (OTUs) for every distance level that can be used to form OTUs. **DOTUR** can use nearest (single linkage), furthest (complete linkage) or average neighbor (UPGMA) algorithms. The default is further neighbor. Once sequences are assigned to an OTU, frequency dat for each distance level is used to construct rarefaction curves, Shannon's and Simpson's diversity indices. Chao1, ACE, Jackknife and Bootstrap richness estimators are also calculated. |
| Homepage | http://www.plantpath.wisc.edu/fac/joh/dotur.html |
| Remote Documentation | http://www.plantpath.wisc.edu/fac/joh/DOTUR/DOTURManual.pdf |
